# Urinary chemerin as a potential biomarker for inflammatory bowel disease

**DOI:** 10.3389/fmed.2022.1058108

**Published:** 2022-11-10

**Authors:** Stefan Gunawan, Tanja Elger, Johanna Loibl, Tanja Fererberger, Stefanie Sommersberger, Arne Kandulski, Martina Müller, Hauke Christian Tews, Christa Buechler

**Affiliations:** Department of Internal Medicine I, Gastroenterology, Hepatology, Endocrinology, Rheumatology, and Infectious Diseases, University Hospital Regensburg, Regensburg, Germany

**Keywords:** urine biomarker, feces, calprotectin, creatinine, C-reactive protein, Crohn’s disease, ulcerative colitis, inflammatory bowel disease (IBD)

## Abstract

**Purpose:**

Systemic levels of the adipokine chemerin are elevated in different inflammatory conditions such as inflammatory bowel disease (IBD). In IBD, chemerin protein expression in colon mucosa is induced and serum chemerin levels are increased. Aim of this study was to identify chemerin protein in human feces and/or urine and to evaluate a possible association with IBD activity.

**Materials and methods:**

Feces and urine of 40 patients with IBD and the respective sera of 34 patients were collected. Chemerin levels were analyzed by immunoblot in feces and urine samples. In addition, enzyme-linked immunosorbent assay (ELISA) was used to measure chemerin in all urine, feces and serum samples of the patients and in urine of 17 healthy controls.

**Results:**

Chemerin was not detectable in 80% of the human feces samples by ELISA. Chemerin in human urine was detected by immunoblot and ELISA. Compared to serum levels, urinary concentration was about 6,000-fold lower. Urinary chemerin did not differ between patients with ulcerative colitis (*n* = 15) and Crohn’s disease (*n* = 25). Urinary chemerin was not related to its serum levels, did not correlate with serum C-reactive protein level and negatively correlated with serum creatinine. Of note, urinary chemerin of patients with a fecal calprotectin > 500 μg/g was significantly higher compared to patients with lower calprotectin levels and compared to healthy controls. Serum creatinine did not differ between the patient groups.

**Conclusion:**

Urinary chemerin might present a novel non-invasive biomarker for monitoring IBD severity and clinical course.

## Introduction

Inflammatory bowel disease (IBD) with the two main entities Crohn’s disease (CD) and ulcerative colitis (UC) is a chronic inflammatory disease with rising prevalence (1–4).

Chemerin is an adipokine and functions as an attractant for immune cells. Moreover, this protein is involved in glucose metabolism, blood pressure homeostasis, and carcinogenesis (5–10). Serum chemerin levels are increased in obesity and various studies proved an association of circulating chemerin with systemic markers of inflammation (11–17).

Chemerin exerts pro- and anti-inflammatory activities. Chemerin as well as chemerin derived C-terminal peptides function as pro-resolving factors (18–21). Chemokine-like receptor 1 (CMKLR1) is the best studied chemerin receptor, so far. G-protein receptor 1 is a further functional chemerin receptor whereas chemokine (C–C motif) receptor-like 2 (CCRL2) has no signaling activity (6, 22, 23). Chemerin and CMKLR1 are abundant in intestinal epithelial cells and their expression in the epithelial barrier is associated with disease severity of IBD (24, 25). CMKLR1 knockout could, however, neither prevent nor improve dextran sulfate sodium (DSS) colitis. Accordingly, intraperitoneal application of recombinant chemerin was without any effect (24). Contrary to this study, it has been demonstrated that exogenous chemerin suppressed the polarization of macrophages from M1 to M2 type and thereby aggravated DSS colitis (25).

Chemerin deficient mice and animals with lack of intestinal epithelial cell CMKLR1 were more sensitive to microbiota-driven colon inflammation. Loss of chemerin-CMKLR1 signaling reduced expression of lactoperoxidase, which is highly abundant in colonic epithelial cells. Lactoperoxidase exerts antimicrobial effects suggesting that chemerin protected from dysbiosis in IBD (26). Interestingly, an antimicrobial activity of chemerin as well as an internal twenty amino acid chemerin peptide in epidermis has also been described (27, 28).

Circulating chemerin was higher in experimental colitis and was increased in serum of patients with CD and UC in comparison to healthy controls (24, 29, 30).

Fecal biomarkers have emerged as tools in diagnosis and monitoring the therapeutic response of IBD. Fecal calprotectin is generally used to monitor intestinal inflammation and to anticipate disease relapse in clinical practice (31, 32). This biomarker is released by granulocytes, and therefore, associated with intestinal inflammation. Thus, it is not specific for IBD (33, 34). Extending fecal calprotectin to include additional biomarkers may improve IBD diagnosis. In consideration that serum and colonic chemerin are higher in IBD (24, 25, 29, 30), fecal chemerin may become a valuable non-invasive biomarker for IBD diagnosis.

Until now, to our knowledge, there are no studies that have examined whether chemerin can be detected in feces. Urine is a further biological fluid becoming increasingly important for biomarker studies and development (35). Serum chemerin is strongly induced in patients with renal dysfunction (36), and impaired renal excretion may contribute to higher circulating chemerin levels. Chemerin protein was indeed detected in urine of rats by enzyme-linked immunosorbent assay (ELISA) (37). Rat urine has a protein content of approximately 1 g/l (38) suggesting that there is about 10 pg chemerin/μl urine. Serum chemerin of the rats was about 40 ng/ml and was 4-fold higher than urinary levels (37).

The aim of the current investigation was to study whether fecal and/or urinary chemerin has the potential to become a diagnostic non-invasive biomarker for IBD.

## Materials and methods

### Patients

Patients with confirmed IBD diagnosis were recruited from the outpatient and inpatient clinic at the Department of Internal Medicine I at the University Hospital of Regensburg from 06.12.2021 to 23.06.2022. IBD was diagnosed based on accepted endoscopic, histologic, and clinical criteria (39, 40). Patients who were pregnant, had known coagulopathy, or were unable to give informed consent were excluded from the study. Moreover, urine of 17 healthy controls was collected. Controls were students, hospital staff and spouse of the patients. Details of the study groups are summarized in Table 1.

**TABLE 1:** Characteristics of the patients and controls.

Characteristics	Patients	Controls
Number (females/males)	40 (16/24)	17 (10/7)
Age (years)	42 (19–67)	42 (23–78)
BMI (kg/m^2^)	25.0 (16–44)	n.d.
CRP (mg/l)	2.7 (0.6–144.0)	n.d.
Creatinine (mg/dl)	0.83 (0.51–1.12)	n.d.
GFR (ml/min)	100 (72–136)	n.d.
First diagnosis (years)	10 (0–42)	n.a.

BMI, Body mass index; CRP, C-reactive protein; GFR, Glomerular filtration rate; n.a., Not applicable; n.d., Not documented.

### Enzyme-linked immunosorbent assay

ELISA to measure human chemerin was from R&D Systems (Wiesbaden, Nordenstadt, Germany; Cat # DY2324). Serum was diluted 1:250 fold for analysis. Urine was centrifuged for 5 min at 4,000 rpm and was used undiluted. Fecal protein was prepared as described below and was used undiluted.

### Immunoblot analysis

Immunoblot was performed as described (41). Urine as well as fecal protein was used undiluted and 16 μl were loaded per lane. Chemerin antibody was from R&D Systems (Cat # AF2324, RRID:AB_416577). Coomassie Brilliant Blue R-250 Staining Solution was from Bio-Rad Laboratories (Feldkirchen, Germany, Cat #1610436). ImageJ was used to quantify protein levels (42).

### Isolation of fecal protein

Fecal homogenates were prepared as described (43). Feces (0.5 to 2.0 g) was homogenized in phosphate buffered saline (PBS) supplemented with protease inhibitor (Protease Inhibitor Cocktail cOmplete™ EDTA-free, Roche Diagnostics, Penzberg, Germany; Cat # 11836170001) by the gentleMACS Dissociator using gentleMACs M-tubes (Miltenyi Biotec, Bergisch Gladbach, Germany; Cat # 130-093-236). One ml aliquots were dried overnight in a vacuum concentrator. The homogenate was dissolved in PBS with protease inhibitor to 2 mg dry weight/ml. Material was stored at −80°C until use.

### Statistical analysis

Data are presented as boxplots. Statistical differences were analyzed by Mann Whitney *U*-test or Kruskal-Wallis Test and associations between two measures were analyzed by Spearman correlation (SPSS Statistics 25.0 program, IBM, Leibniz Rechenzentrum, München, Germany), and a value of *p* < 0.05 was regarded significant.

## Results

### Chemerin is rarely detectable in feces

**FIGURE 1:**
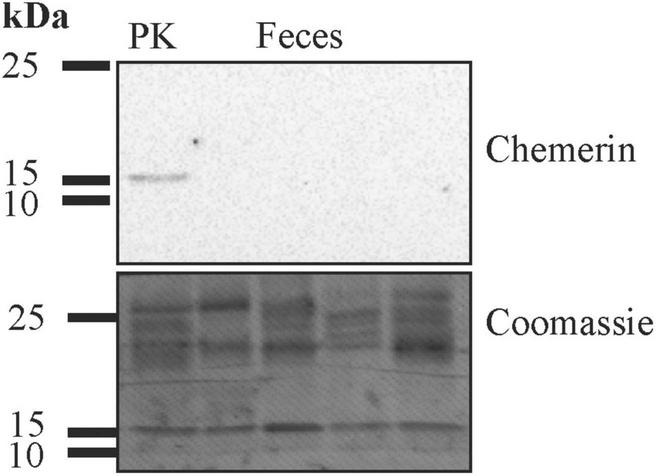
Immunoblot of feces for analysis of chemerin protein expression. After the experiment the membrane was stained with Coomassie Brilliant Blue R-250. The molecular weight size markers are given. Human liver lysate was used as positive control (PK).

Protein isolated from human feces was used for immunoblot analysis. Chemerin could not be detected in feces of six patients analyzed by this method (Figure 1 and data not shown). ELISA was used to measure fecal chemerin of 40 patients. Fecal chemerin levels of 80% of the patients were below the detection limit of the assay with a range from 31.2 to 2,000 pg/ml chemerin. The eight patients with fecal chemerin levels above 0 had a low median level of 35.0 (8.8–156.7) pg/ml. Fecal chemerin was not related to fecal calprotectin levels (*p* = 0.533).

### Chemerin is detectable in urinary samples

**FIGURE 2:**
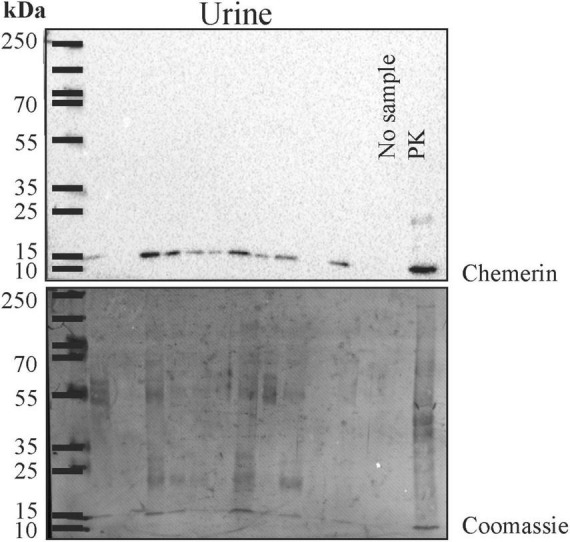
Immunoblot of urine for analysis of chemerin protein. After the experiment the membrane was stained with Coomassie Brilliant Blue R-250. The molecular weight size marker is shown and the respective molecular weights are given. Human liver lysate was used as positive control (PK).

Human urine was used for immunoblot analysis and chemerin was detected in twenty-one of the twenty-four samples (Figure 2 and data not shown). Urinary chemerin had a median expression of 13.4 and ranged from 0 to 51.0.

**FIGURE 3:**
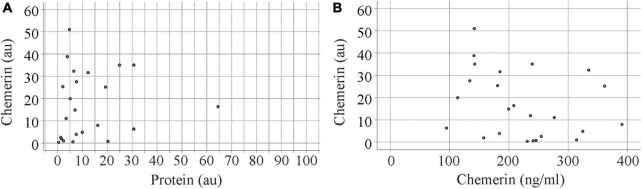
Correlation analysis of urinary chemerin protein with total protein levels and serum chemerin. **(A)** Correlation of urinary chemerin protein determined by immunoblot and total protein in urine. Data of one patient is not shown in the figure because of the high protein content of this urine sample. **(B)** Correlation of urinary chemerin protein determined by immunoblot and serum chemerin measured by enzyme-linked immunosorbent assay (ELISA).

After the immunoblot experiment, total urinary protein on the membrane was stained with Coomassie Brilliant Blue R-250 and quantified using ImageG (42; Figure 2). Urinary chemerin protein levels were not correlated with total urinary protein concentrations (*r* = 0.262, *p* = 0.217; Figure 3A). Urinary chemerin levels as determined by immunoblot did not correlate with serum chemerin measured by ELISA (*r* = −0.336, *p* = 0.109; Figure 3B).

### Urinary chemerin concentrations in relation to serum chemerin, gender, BMI, and serum creatinine

Enzyme-linked immunosorbent assay (ELISA) was used to quantify urinary chemerin protein of 40 patients with IBD. Median chemerin protein in urine was 34 (20–1,470) pg/ml. Serum of 34 of these patients was available, and in serum chemerin was 190 (82–391) ng/ml. Thus, urinary chemerin expression was about 6,000-fold lower than serum levels. There was no correlation between urinary and serum chemerin levels (*r* = −0.095, *p* = 0.593).

**FIGURE 4:**
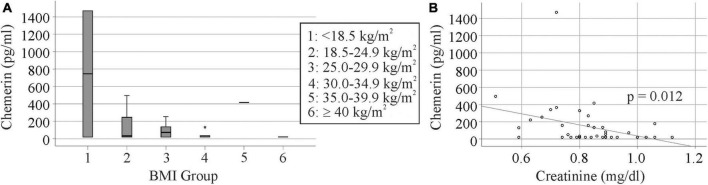
Urinary chemerin levels in relation to BMI and serum creatinine. **(A)** Urinary chemerin stratified for BMI. The asterisk marks an outlier (three box lengths from the median). **(B)** Correlation of urinary chemerin with serum creatinine.

Urinary chemerin levels did not differ between females and males (*p* = 0.633). Age (*r* = 0.023, *p* = 0.887) and body mass index (BMI; *r* = −0.191, *p* = 0.250) did not correlate with urinary chemerin concentration. When patients were stratified for BMI there were two patients with a BMI < 18.5 kg/m^2^, 20 patients with a BMI between 18.5 and 24.9 kg/m^2^, nine patients with a BMI between 25.0 and 29.9 kg/m^2^, seven patients with a BMI between 30.0 and 34.9 kg/m^2^, one patient with a BMI between 35.0 and 39.9 kg/m^2^, and one patient with a BMI > 40 kg/m^2^. Urinary chemerin expression did not significantly differ between these groups (*p* = 0.330) (Figure 4A).

Urinary chemerin expression negatively correlated with serum creatinine (Figure 4B) whereas the association with glomerular filtration rate was not significant (*r* = 0.287, *p* = 0.073).

Serum chemerin did neither correlate with serum creatinine (*r* = 0.045, *p* = 0.799) nor glomerular filtration rate (*r* = 0.086, *p* = 0.627).

### Urinary chemerin in relation to fecal calprotectin and serum C-reactive protein

**FIGURE 5:**
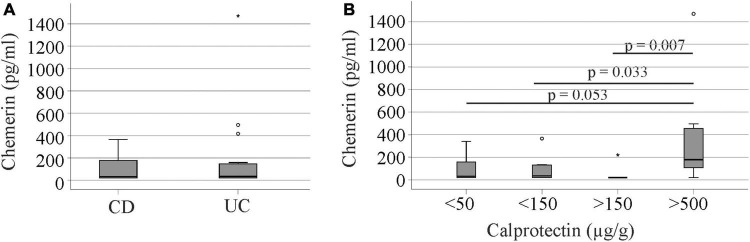
Urinary chemerin protein in Crohn’s disease (CD) and ulcerative colitis (UC) patients and in relation to fecal calprotectin. **(A)** Urinary chemerin of CD and UC patients. **(B)** Urinary chemerin protein stratified for fecal calprotectin levels. Circles (1.5 box lengths from the median) and asterisks (three box lengths from the median) mark outliers.

In the group of 40 IBD patients, 25 patients had been diagnosed with CD and 15 patients with CU. Urinary chemerin levels were comparable between the two groups (Figure 5A).

There was no correlation between urinary chemerin concentration and fecal calprotectin levels (*r* = 0.117, *p* = 0.417). Therefore, we analyzed patient cohorts divided according to their fecal calprotectin levels. Seventeen patients had calprotectin levels below 50 μg/g, 10 patients had calprotectin levels from 50 to 149 μg/g, 6 patients had calprotectin levels from 150 to 500 μg/g and seven patients calprotectin levels > 500 μg/g.

Patients with fecal calprotectin < 50 μg/g were the oldest and had the lowest glomerular filtration rate (GFR). CRP was highest in patients with calprotectin > 500 μg/g (Table 2). Gender distribution, BMI, creatinine, and time since first diagnosis were similar between the groups (Table 2).

**TABLE 2:** Characteristics of the patients stratified for fecal calprotectin levels.

Calprotectin μg/g	< 50	< 150	> 150	> 500	*P*-value
Number (females/males)	17 (8/9)	10 (5/5)	6 (2/4)	7 (1/6)	
Age (years)	53^a,b,c^ (30–67)	34^b^ (23–55)	29^c^ (19–56)	28^a^ (20–65)	0.038^a^ 0.010^b^ 0.013^c^
BMI (kg/m^2^)	26 (20–44)	24 (17–35)	25 (22–32)	22 (16–40)	
CRP (mg/l)	1.9^a^ (0.6–21.1)	1.2^b^ (0.6–26.3)	6.7^c^ (0.6–18.1)	25.0^a,b,c^ (11.2–144.0)	0.001^a^ 0.001^b^ 0.025^c^
Creatinine (mg/dl)	0.84 (0.67–1.06)	0.78 (0.59–1.12)	0.86 (0.63–1.03)	0.80 (0.51–1.06)	
GFR (ml/min)	89^a,b,c^ (72–119)	105^b^ (91–120)	106^c^ (95–131)	110^a^ (97–136)	0.007^a^ 0.020^b^ 0.025^c^
First diagnosis (years)	14 (2–42)	10 (0–32)	6 (0–35)	3 (1–16)	
Chemerin pg/ml	32.9 (20.0–341.7)	35.2^a^ (19.9–365.7)	20.1^b^ (20.1–220.8)	178.3^a,b^ (19.8–1470.3)	0.033^a^ 0.007^b^
Chemerin CV%	113	127	153	126	

Median values and ranges are listed. Significant different measures were marked with identical uppercase letters (BMI, Body mass index; CV, Coefficient of variation; CRP, C-reactive protein; GFR, Glomerular filtration rate).

Patients with high fecal calprotectin > 500 μg/g had higher urinary chemerin levels in comparison to the three other groups with similar levels (Figure 5B). Urinary chemerin protein varied within the groups (11.0 to 18.4-fold in patients with fecal calprotectin < 500 μg/g) and the variation was 4 to 6-fold higher in patients with fecal calprotectin > 500 μg/g. The coefficient of variation (CV%) is a measure of relative variability and did not markedly differ between the groups (Table 2).

C-reactive protein (CRP) as a serum marker for inflammation correlated positively with fecal calprotectin in our cohort (*r* = 0.516, *p* = 0.001) but there was no significant association of urinary chemerin and serum CRP (*r* = 0.284, *p* = 0.080).

Serum chemerin levels positively correlated with CRP (*r* = 0.321, *p* = 0.064) though this association was not significant. Serum chemerin concentrations did not correlate with fecal calprotectin (*r* = 0.124, *p* = 0.484) and levels did not differ between patients stratified for fecal calprotectin levels (*p* = 0.224).

Histologic remission was documented for 32 patients and was achieved in nine patients. Urinary chemerin did not differ between these groups (*p* = 1.000). When patients with fecal calprotectin > 500 μg/g were excluded, urinary chemerin levels were still similar between patients with and without histologic remission (*p* = 0.215).

### Urinary chemerin concentrations of healthy controls

**FIGURE 6:**
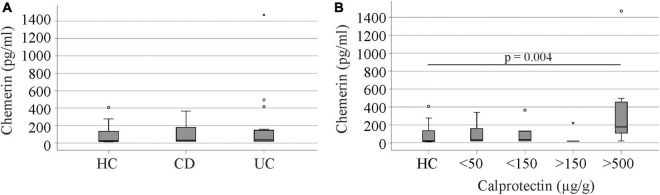
Urinary chemerin protein of healthy controls (HC), Crohn’s disease (CD), and ulcerative colitis (UC) patients. **(A)** Urinary chemerin of HC, CD, and UC patients. **(B)** Urinary chemerin protein of HCs and patients stratified for fecal calprotectin levels. Circles (1.5 box lengths from the median) and asterisks (three box lengths from the median) mark outliers.

Urinary chemerin protein of 17 healthy controls was also measured. Gender distribution and age of controls and patients were comparable (Table 1). The 10 female and 7 male controls had similar levels of chemerin in urine (*p* = 0.813). Urinary chemerin did not correlate with age (*r* = 0.130, *p* = 0.618) Urinary chemerin protein did not differ between the controls, patients with CD or UC (Figure 6A). Controls had urinary chemerin levels similar to the patients stratified for calprotectin levels with the exception of the group with fecal calprotectin > 500 μg/g. Urinary chemerin protein of this group was significantly higher compared to the controls (Figure 6B).

### Urinary chemerin in relation to complications and the current inflammatory bowel disease medication

There were 17 patients who suffered from extra-intestinal manifestations of IBD and seven patients with fistulas. Urinary chemerin did not differ in patients with and without these complications (*p* = 0.812 and *p* = 0.707, respectively).

Common medications used to treat IBD were anti-tumor necrosis factor monoclonal antibodies (10 patients), anti-p40 monoclonal antibodies (12 patients), mesalazine (15 patients) and corticosteroids (10 patients). Urinary chemerin of patients taking anti-tumor necrosis factor monoclonal antibodies (*p* = 0.548), anti-p40 monoclonal antibodies (*p* = 0.079), mesalazine (*p* = 0.192) and corticosteroids (*p* = 0.914) was similar to patients without these medications.

## Discussion

This is to our knowledge the first study to detect chemerin protein in human urine samples. Urinary chemerin protein levels were higher in patients with IBD and fecal calprotectin > 500 μg/g compared to patients with lower levels and healthy controls. Thus, urinary chemerin levels may be discussed as a novel non-invasive marker for intestinal inflammation in patients with IBD.

It has been described that serum chemerin is elevated in patients with impaired renal clearance (36, 44). In the general population serum chemerin levels were inversely associated with renal function (45). We were able to detect chemerin protein in human urine samples and could show that urinary chemerin was inversely correlated with serum creatinine. This suggests that serum chemerin is partly cleared from the body by renal elimination. Urinary chemerin concentration is low and about 6,000-fold less than serum levels. A rough estimate for male rats was a 4-fold difference between serum and urine (37) suggesting that renal chemerin excretion greatly differs between humans and rats. Urinary volume of humans is about 2 l per day (46) and about 70 ng chemerin may be excreted by the kidneys. To get exact numbers it has to be clarified whether degraded chemerin, which is no longer detected by the ELISA, is also present in human urine.

Immunoblot analysis revealed that chemerin protein in urine has a molecular weight of about 15 kDa suggesting that at least part of the chemerin protein is not degraded. Chemerin in human serum is mostly inactive and C-terminal cleavage of a few amino acids produces biologic active variants (6, 47). Activation of chemerin is achieved by several proteases including the inflammatory serine proteases tryptase, plasmin and elastase (48). So further studies should be designed to investigate which isoforms of chemerin are abundant in urine.

Also noteworthy, high inter-individual variations of urinary chemerin protein levels can be detected. Whereas serum chemerin of the patients studied varied about 5-fold (CV% 39), urinary chemerin varied about 70-fold in the whole cohort (CV% 176) and about 20-fold (CV% 123) when patients with calprotectin > 500 μg/g were excluded.

Urinary chemerin was not correlated to serum chemerin, total urinary protein content, gender, BMI or age. There was a negative correlation with serum creatinine with an about 2-fold variation, which thus cannot explain the wide range of urinary chemerin protein levels. Future studies are needed to characterize the mechanisms, which contribute to urinary chemerin concentrations.

It is important to note that fecal calprotectin ranged from 18 to 1,616 μg/g in the whole cohort and from 18 to 347 in patients with a calprotectin level < 500 μg/g. This corresponds to an about 19-fold variation in this cohort and an about 90-fold variation in the whole study group. Similar variations of fecal calprotectin in patients with IBD have been reported by others (49, 50). The CV% for calprotectin was 166% in our group and was reported to range from 48% up to 182% in other IBD cohorts (51, 52). Thus, the CV% of 176 for urinary chemerin in the patient cohort is comparable to CV% for fecal calprotectin.

Chemerin and CMKLR1 are expressed in intestinal epithelial cells and are related to local inflammation (24, 25). In contrast to this knowledge, chemerin protein was not detectable in human feces by immunoblot analysis, and was only measurable in stool of 20% of the patients by ELISA. This might be due to low biliary elimination of chemerin and/or degradation of chemerin during fecal excretion.

Interestingly, urinary chemerin was increased in IBD patients with high fecal calprotectin. The median urinary chemerin levels of patients with low fecal calprotectin was 33 pg/ml and of patients with levels > 500 μg/g calprotectin was 180 pg/ml. Serum creatinine did not differ between these two groups excluding it as a confounding factor. The CV% of urinary chemerin levels did not differ too much between the calprotectin groups.

Urinary chemerin did not increase in parallel with calprotectin levels and did not correlate with CRP suggesting that it is not simply a marker of inflammation. Although the cause for higher urinary chemerin is unknown it may serve as an additional disease activity biomarker in IBD.

Urinary chemerin was comparable between healthy controls and IBD patients with calprotectin levels below 500 μg/g. This illustrates that urinary chemerin of the patients does not increase until they develop severe IBD.

Disease activity in IBD was found associated with a higher urinary albumin (53). The etiology of microalbuminuria in patients with IBD remains unclear and is supposed to be a consequence of the acute phase response (53). Higher urinary chemerin in patients with severe IBD may thus reflect renal impairment. This suggests that urinary chemerin may be analyzed for its suitability as a biomarker for renal dysfunction in IBD.

The current study cohort was rather small and relation of urinary chemerin with disease severity, progression or remission has to be assessed in larger study groups. Whether urinary chemerin may be useful to discriminate active IBD from intestinal inflammation caused by infections, specific drugs, cancer, or diverticulitis needs further analysis.

Urinary chemerin did not differ between patients with CD or UC. It could not discriminate patients with low from patients with medium fecal calprotectin levels; nor patients with histologic remission from patients without histologic remission. Therefore, chemerin protein in urine is not of diagnostic value in this regard but may be useful as an additional biomarker to fecal calprotectin to monitor IBD disease activity. A novel urine biomarker for IBD is highly desirable for clinical application as a follow-up and disease-activity marker because it can be collected recurrently by non-invasive techniques.

Limitation of this work is the small number of patients analyzed, and thus possible differences of urinary chemerin between CD and UC patients with high fecal calprotectin could not be analyzed. Moreover, urinary chemerin was not determined during therapy to monitor response to treatment.

To summarize, present study detected chemerin in human urine and showed that urinary chemerin levels of IBD patients with high fecal calprotectin were increased. Urinary chemerin is a potential novel and easily accessible biomarker for the monitoring of IBD patients.

## Data availability statement

The original contributions presented in this study are included in the article/supplementary material, further inquiries can be directed to the corresponding author.

## Ethics statement

This study was approved by the Ethics Committee of the University Hospital of Regensburg (Protocol No. 19-1309-101, Approval date: 20.02.2019) and all participants gave written informed consent to the study. The study was performed according to the updated guidelines of good clinical practice and updated Declaration of Helsinki.

## Author contributions

CB, AK, and HT: conceptualization. TF, SS, HT, TE, SG, and JL: resources. SG: investigation. CB: statistical analysis and writing—original draft preparation. All authors: writing—review and editing, critically revised the manuscript, approved the final version to be published, and agree to be accountable for all aspects of the work.
